# Stem Cells for Cardiac Repair: Status, Mechanisms, and New Strategies

**DOI:** 10.4061/2011/310928

**Published:** 2011-06-15

**Authors:** Ren Mingliang, Zhang Bo, Wang Zhengguo

**Affiliations:** Department 4, State Key Laboratory of Trauma, Burn and Combined Injury, Research Institute of Surgery and Daping Hospital, Third Military Medical University, Chongqing 400042, China

## Abstract

Faced with the end stage of heart disease, the current treatments only slow worsening of heart failure. Stem cells have the potential of self-renewal and differentiation. It is expected to replace and repair damaged myocardium. But many clinical trials have shown that the stem cell therapy of heart failure is modest or not effective. The possible causes for the limited effects of stem cell in curing heart failure are the stem cells which have been transplanted into the ischemic heart muscle may suffer low survival rate, affected by inflammatory molecules, proapoptotic factor, and lack of nutrients and oxygen, and then the stem cells which home and have been completely transplanted to the site of myocardial infarction become very small. Therefore, through preconditioning of stem cells and appropriate choice of genes for mesenchymal stem cell modification to improve the survival rate of stem cells, ability in homing and promoting angiogenesis may become the newly effective strategies for the application of stem cells therapy in heart failure.

## 1. Introduction

 With the changes in lifestyle and aging of population, the morbidity of hypertension, coronary heart disease, and other common cardiovascular disease has shown a continuous rising tendency. As the end stage of cardiovascular disease, heart failure suffers high morbidity and poor prognosis. Heart failure was mentioned on 277 193 death certificates and was the underlying cause in 56 565 of those deaths in the United States in 2007 [1, 2]. According to a random sampling survey to 15,518 residents aging from 35 to 74 in China in 2003, the prevalence of heart failure was 0.9%. It was lower than developed countries. However, the total prevalence was still up to 4,000,000, and the incidence was increasing ceaselessly [3]. Current drug treatment can only improve symptoms without preventing the ventricular remodeling and the deterioration of progressive heart function. Heart transplantation is an effective means of treating patients with heart failure. But the vast majority of patients are restricted by the age, the donor, surgical complications, medical costs, and so forth. 

Stem cells are the origin cells of various mature cells. They have the potential of self-renewal and differentiation. Either immediately after isolation or after expansion in vitro, stem cells are transplanted into a specific region of the heart, and ultimately replace, repair the myocardial necrosis or pathological cells; then the aim of curing heart failure can be achieved and it has brought a bright prospect for the treatment of heart failure. Although the basic research on the differentiation of stem cell transplantation has not yet achieved consistent results, many clinical trials regarding the stem cell transplantation for acute and chronic heart failure have been carried out [4]. The results suggest that stem cells therapy improve the clinical symptoms modestly, but almost have no effect in preventing ventricular remodeling and long-term prognosis.

## 2. Clinical Experience

**Figure 1:**
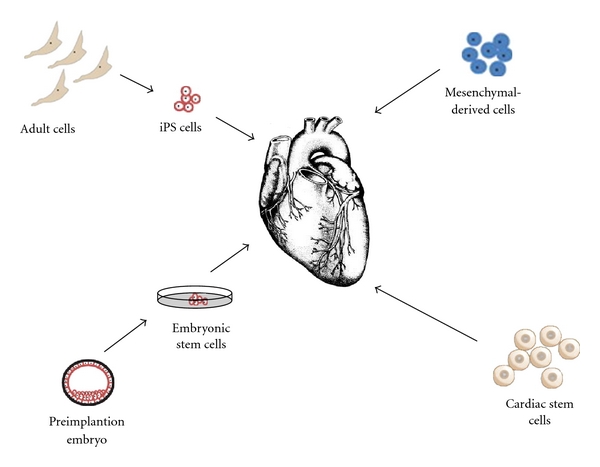
The cell types used in heart repair.

Currently, the main types of stem cells used for clinical treatment include bone marrow-derived cells (BMCs), adipose-derived stem cells (ADSCs), cardiac stem cells (CSCs), peripheral blood derived cells, embryonic stem cells (ES), and induced pluripotent stem cells (Figure 1). Autologous skeletal myoblast is one of the stem cells which have been firstly used in myocardial regeneration. It can counteract ischemia and has the ability of regeneration after damage. However, skeletal myoblasts cannot form an effective electrical coupling synchronization with the living myocardial cells and may lead to malignant arrhythmia [5]. It has been shown that ES transplantation can improve cardiac contractile function and still have proliferative capacity. However, ethical debate of embryonic stem cells, immune rejection, and the risk of tumorigenicity still hinder its application. 

### 2.1. Bone Marrow-Derived Cells

 BMC is currently the most widely used cells in clinical trials (Table 1). It mainly contains mesenchymal stem cells (MSCs), hematopoietic stem cells, border cells, endothelial progenitor cells, and so forth. Although, the acquisition of BMC induces pain and the number of BMCs decreases dramatically with age, it is relatively simple to separate and large numbers of cells can be obtained without the need for ex vivo expansion. It provides initial cells with many mixed components and mutidifferentiation potential. Furthermore, transplantation of autologous cells avoids immune rejection and ethical disputes caused by embryonic stem cells transplantation. REPAIR-AMI [6] multicenter randomized controlled trial included 204 patients with acute myocardial infarction. After 3~7 days' reperfusion therapy, they received the autologous bone marrow-derived mononuclear cells (BMMNCs) intracoronary or placebo (medium) treatment. 4 months later, the treatment group left ventricular ejection fraction (LVEF) improved slightly. Compared with the control group, there were statistically significant differences. However, in one-year-followup, the cardiac indicators associated with remodeling had no significant changes. Subgroup analysis showed that only patients with large myocardial infarction had a marked effect. European STAR-heart [7] examined the stem cells treatment for chronic heart failure. 391 patients with chronic heart failure patients due to ischemic heart disease (myocardial infarction) were included, diagnostic criteria for LVEF *⩽* 35%. BMMNCs directly infused to related artery infarction through angioplasty balloon catheter. Effects on the patients were evaluated after treatment of 3 months to 5 years. LVEF, cardiac index, exercise capacity, oxygen uptake, and left ventricular contractility had improved significantly. In addition, in the first assessment after 3-month treatment the beneficial effects observed lasted to 12 months and 60 months, while there was a phenomenon of deterioration of left ventricular function in the control group at the same time. Compared with the control group, long-term mortality of patients treated with stem cells significantly decreased.

Although the results of many experiments show that BMC transplantation can improve LVEF (compared with the control group, only about 3%), there are no obvious long-term effects [17]. Recent meta-analysis of a series of published clinical trials before 2009 also found that although coronary artery transplanted stem cells can significantly reduce left ventricular end systolic volume in patients with acute myocardial infarction, they have no significant effect on left ventricular end-diastolic volume [19]. In the randomized controlled BOOST trial [12], patients with ST segment elevation after acute myocardial infarction received an intracoronary BMC infusion. Results showed that LVEF had improved after 6 months, but there were no significant differences compared with the control group after 18 months. In 5-year followup, all indicators did not find differences with the control group. However, according to the current trials, the rate of adverse effect with autologous BMC transplantation is relatively low, and compared with conventional treatment, the incidence of malignant arrhythmias, restenosis did not significantly elevate. The safety of BMC transplantation has been initially verified. 

Clinical studies on intracoronary BMC infusion in patients with myocardial infarction revealed mixed results, partly because of marked heterogeneity between trials [12]. The preclinical studies were performed in young healthy animals with a single coronary occlusion. The clinical trials were performed in old patients with extensive comorbidities. The numbers and proliferation of stem cells decline in elderly patients. This may be the reason that the clinical trials were not as beneficial as the animal studies. But a meta-analysis suggests that BMC therapy is likely more effective in ageing and diabetic individuals [20]. A speculation is that the patients who are ageing, postmenopausal female, or diabetic are likely to suffer from impaired endothelium and to have inadequate physiological angiogenesis response to ischemia, therefore tend to gain beneficial effects from the supplementation of BMC. Many current clinical trials are not double-blind studies, and there could be a “placebo effect”. The long-term effectiveness of BMC treatment of heart failure needs the evidence of large-scale double-blind randomized controlled trials.

### 2.2. Cardiac Stem Cells

Many kinds of CSCs which were taken from adult heart tissue can be isolated and identified including c-kit+ cells and Sca-1+ cells [21]. Regardless of the gender or age of the patient, or of diabetes, David et al. [22] isolate in all of them a pool of functional CSCs. Although less so in older or diabetic patients, they found that cells had long telomeres, or “caps,” on their chromosomal ends indicating that expanded CSCs retained a significant growth reserve. CSCs can differentiate into three kinds of major heart cell precursors: myocardial cells, smooth muscle cells and epithelial cells [23]. Although isolation of these cells requires access to cardiac tissue, CSCs have been successfully isolated from right ventricular endomyocardial biopsy [24], a technique that could be used clinically. Some Phase I clinical trials are being conducted to observe the safety and feasibility of using CSCs in patients (http://www.clinicaltrials.org/NCT00474461).

### 2.3. Induced Pluripotent Stem Cells

In 2006, Takahashi and Yamanaka [25] reported the first successful reprogramming by delivering four stem cell-related genes (Oct3/4, Sox2, Klf4, c-Myc) into skin fibroblasts. The adult cells conversed into an ES-like characteristic of pluripotent stem cells, called induced pluripotent stem cells (iPSs). iPSs avoid a moral controversy, and can be patient-specific stem cells. Human iPSs have been implanted in mouse models of myocardial infarction, and it was indicated that they regenerated myocardium, smooth muscle, and endothelial tissue, restoring postischemic contractility performance and electric stability [26]. As with ES, iPSs-derived cells may be contaminated with potentially tumorigenic cells. Ieda et al. [27] reported that a combination of three developmental transcription factors rapidly and efficiently reprogrammed postnatal cardiac or dermal fibroblasts directly into functional cardiomyocytes. Reprogramming of endogenous or explanted adult cells might provide a source of cardiomyocytes for regenerative approaches.

### 2.4. Adipose-Derived Stem Cells

Numerous studies have provided evidence that ADSCs contain a population of adult multipotent mesenchymal stem cells and endothelial progenitor cells. The similarities between bone marrow-derived cells and the ADSCs suggest the potential of the adipose tissue to act as an alternative, and perhaps preferable, cell source for repairing damaged myocardium. ADSCs are able to differentiate into multiple cell lineages including cardiomyocytes [28]. ADSCs can effectively improve LVEF in animal models of acute and chronic myocardial infarction. According to a small, first-of-its-kind study [29], ADSCs can be safely obtained and infused inside the hearts of patients following an acute heart attack. ADSCs are emerging as a new source of adult stem cells for cardiovascular repair. Certainly, more clinical trials are needed to demonstrate the long-term efficacy and safety.

## 3. Mechanism of Stem Cells Therapy

The stem cells including ES, CSCs, and iPSs can be differentiated into cardiomyocytes after transplantation and restore contractile function. They also can be differentiated into endothelial cells and promote angiogenesis, turn part of the damaged heart muscle alive, and limit scar expansion. Stem cells including BMC and ADSCs transdifferentiate into cardiomyocytes in vivo, but no one has yet observed that MSCs give rise to fully differentiated and functional cardiomyocytes in vivo [30, 31]. With the discovery of paracrine effect of the stem cell, many studies have confirmed that stem cell therapy of heart failure depends on the mechanism, mainly in the promotion of angiogenesis, against myocardial apoptosis, immune regulation, and so on [32]. 

(1) The autocrine or paracrine growth factor such as vascular endothelial growth factor (VEGF) promotes reconstruction of myocardial vascular network [33]. VEGF can increase permeability of capillary wall, activate matrix metalloproteinase, and promote endothelial cell proliferation and migration. It is one of the most important angiogenesis factors. Research has shown that sustained high expression of VEGF, cooperated with the other angiogenesis factors (such as bFGF), may promote the formation of smooth muscle cells, participate in the “arteriogenesis” process, and improve myocardial ischemia. Tang et al. [34] confirmed capillary proliferation in the areas of acute myocardial infarction and surrounding area after stem cell transplantation.

(2) MSCs transplantation inhibits the activation of NF-*κ*B, attenuates the protein production of TNF-*α* and IL-6, and increases anti-inflammatory cytokines IL-10 expression [35]. As proinflammatory cytokines, TNF-*α* and IL-6 have a toxic effect on myocardial cells, can inhibit the cardiac contractile function, and induce apoptosis of cardiomyocyte. In addition, they can regulate the expression of monocyte chemoattractant protein, vascular endothelial cellular adhesion molecule to chemotaxis of inflammatory cells into myocardial tissue, increase myocardial tissue inflammatory responses, and thus promote the progress of ventricular remodeling after AMI [36]. As an anti-inflammatory cytokine, IL-10 may be expressed by monocytes, macrophages, cardiac cells, and so on. Through inhibition of NF-*κ*B activity to decrease TNF-*α* and IL-6 expression, it can also inhibit the inflammatory response to some degree [37].

(3) Cardiomyocyte hypertrophy and the extracellular matrix deposition play major roles in the remodeling of noninfarcted myocardium. Pathologic increase in extracellular collagen leads to interstitial fibrosis, and although this can be useful in limiting ventricular enlargement, it decreases the compliance of ventricular wall and affects heart function [38]. MSCs transplantation improves cardiac function in part through regulation of cardiac fibroblasts proliferation and transcriptional downregulation of types I and III collagen syntheses [39]. This may be one of the mechanisms through which they inhibit the ventricular remodeling.

## 4. New Strategy and Direction of Stem Cell Therapy for Heart Failure

The stem cell transplantations for treatment of heart failure have modest effect or no effect. In addition to problems in the best method of cell delivery, the treatment time, and patient selection, an important common issue the transplantation of stem cells faces is that the survival rate in the host is very low. Because they are transplanted in an ischemia, hypoxia, and proapoptotic niche, most stem cells cannot survive after transplantation [40]. The results of real-time PCR and TUNEL staining after the stem cells transplant showed that more than 90% of the stem cells die within 24 hours after transplantation [41]. Another reason is the amount of cells which migrate to regions of myocardial infarction is too small. PET showed that only 1.3% to 2.6% of the ^18^F-FDG-labeled stem cells which were intracoronary injected migrated to the myocardium 2 hours after injection, while the majority of the cells moved to the tissue outside heart muscle, including liver, spleen, lung, bladder, and brain [42]. 20 hours later, the stem cells settled in the myocardium are only about 1.49% [43]. In response to these problems, there are some new strategies now.

## 5. Preconditioning

In order to improve the viability of stem cells after transplantation and counteract the hypoxia-induced apoptosis, it is an effective protection strategy to use various methods of preconditioning of stem cells before transplanting the cells into the damaged myocardium. Hypoxic preconditioning indicates that the cell is cultivated under hypoxic conditions before transplantation. Hypoxic preconditioning has been found to be able to start the PI3K/AKT signaling pathway and enhance the stability of HIF-1 to increase the antiapoptotic ability of MSCs [44]. Hypoxic preconditioning of peripheral blood mononuclear cells increased the expression of various genes related to antioxidant and survival signals remarkably [45]. Hypoxic preconditioning also enhances the benefit of CSCs therapy for treatment of myocardial infarction by SDF/CXCR4 axis [46]. In addition to hypoxic preconditioning, with some growth factors preconditioning on MSCs, it can improve the ability of cell resistance to apoptosis, too. By using stromal cell-derived factor-1 (SDF-1*α*) to precondition MSCs, Pasha et al. [47] found that SDF-1 preconditioning through SDF/CXCR4 activated multiple signaling pathways, including the PI3K/AKT signaling pathway. Via using MSCs with SDF-1 preconditioning for the treatment of myocardial infarction in rats, it is found that the viability of MSCs which were transplanted significantly increased, and they got a better effect of myocardial repair. By Using diazoxide to precondition MSCs, Afzal et al. found that diazoxide preconditioning can enhance protective role of the MSCs through the NF-*κ*B signaling pathway [48].

## 6. Combination Drug Therapy

 After cell transplantation, administration of drugs combination therapy can also improve the viability of MSCs, too. Statins used to reduce blood lipids in the past were considered to have good protective action. Yang et al. [49] used simvastatin to conduct the combination therapy, and Xu et al. [50] conducted the combination therapy with lovastatin. They both got remarkable curative effects. Then they thought that simvastatin and lovastatin could have a cytoprotective effect by inhibiting the mitochondrial apoptotic pathway to activate the signaling pathway of PI3K/Akt and ERK1/2. In addition, Zhang et al. [51] found that Chinese herbs Berberine can inhibit the hypoxia-induced apoptosis in vitro. The mechanism is also related to the inhibition of the signaling pathway of mitochondrial apoptosis.

## 7. Gene Modified Stem Cells

### 7.1. Increase the Capacity of Antiapoptotic

The early apoptosis of the majority of stem cells after transplantation into the ischemic heart imposes restrictions on their repair functions. Li et al. [52] applied anti-apoptotic gene Bcl-2 to modify MSCs and found that the anti-apoptotic ability of modified MSCs increased; they can also promote VEGF secretion under the hypoxic condition; the number of surviving cells after transplantation in vivo significantly increased, and improvement in cardiac function appeared significantly. Lim et al. [53] modified MSCs with Akt and found that Akt-MSCs tolerated more about hypoxia-induced apoptosis. And after hypoxia, extracellular signal regulated ERK activation, VEGF expression increased, and survival of Akt-MSCs increased after transplantation. Compared with MSCs transplantation alone, it further repaired the damaged myocardium and improved cardiac function. Through enhancing the MSCs's ability of antihypoxia, it can also play the role of increasing the survival rate of the transplanted cells. Tang et al. [40] modified MSCs with heme oxygenase -1 (HO-1), and found tolerance of modified MSCs to hypoxia significantly increased, and the survival rate in the ischemic heart also significantly increased. Seven days after the transplantation, survival rate of HO-1-modified MSCs was as 5 times as that of the control group in vivo. With the modified MSCs transplantation therapy of myocardial infarction, cardiac function was further improved. These genes enhance the viability of MSCs from such perspectives as anti-apoptotic, promoting survivals, antioxidant protection, and so forth.

### 7.2. Promoting Angiogenesis

To promote angiogenesis effectively and improve myocardial blood flow may be one of the effective ways of the treatment of ischemic heart disease. Genes related to angiogenesis include VEGF, Ang-1, FGF-2, IGF, and hepatocyte growth factor (HGF). Yang et al. [54] used liposome-mediated method transfected pcDNA-hVEGF to rat MSCs and used intramyocardial injections method to inject the myocardium of the rat two weeks after myocardial infarction. After four weeks, the results showed that the cardiac function, infarct size, and angiogenesis of the VEGF modified group were significantly better than the other groups, MSCs treatment group. Four weeks after infarction, Yang et al. [55] transplanted HGF-modified MSCs through the non-infarct-related coronary artery into pig heart. The results showed a significant increase in angiogenesis. Sun et al. [56] intramyocardially injected human Ang1-modified MSCs (hAng1-MSCs) to treat rats acute myocardial infarction. The results showed that hAng1-MSCs could survive in the local and express hAng-1 mRNA and protein. Vascular density of hAng1-MSCs and MSCs group was significantly higher than PBS control group, ventricular remodeling and cardiac function were significantly improved. What is more, compared with MSCs, the increase of angiogenesis and arteriogenesis and the decrease of the infarct size and thickening of the left ventricular wall were more significant in hAng1-MSCs group.

### 7.3. Promote Migration

Most of the transplanted stem cells cannot effectively home in on the damaged heart; how to improve the migration of stem cells is one of the research directions in recent years. A series of signals caused by necrosis after myocardial infarction leads to their own MSCs mobilization into peripheral blood pool. The damaged tissue expressed specific receptor or ligand, to guide corresponding stem cells to move and adhere to the injury. SDF-1/CX2CR4 is currently known to promote the homing of MSCs in on the injured tissue [57]. SDF-1/CXCR4 cannot only promote the transplanted MSCs to migrate to the damaged tissue, but also inhibit apoptosis of MSCs, increase the survival rate and proliferation of MSCs [58], and promote the homing efficiency of MSCs from many aspects. 24 hours after the rat coronary artery occlusion-reperfusion, Cheng et al. [59] transplanted retrovirus-mediated CXCR4-modified MSCs (CXCR4-MSCs) by intravenous injection. Results found that the amount of CXCR4-MSCs's homing in on the infarcted myocardium was far more than that of the control group, and 30 days after transplantation, cardiac function and ventricular remodeling indicators gained the upper hand of the MSCs or saline transplantation group.

### 7.4. Anti-Inflammatory

The nonspecific inflammation of the body is one important reason that causes loss of transplanted cells [60]. Tumor necrosis factors (TNF) are an important kind of inflammatory mediators and act as a major factor to the mediated apoptosis of the receptor. Bao et al. [61] studied the anti-inflammatory and cardiac function improvement effects of TNF receptor- (TNFR-) modified MSCs transplantation in the treatment of myocardial infarction. Two weeks after transplantation of TNFG-transfected MSCs into the ischemic myocardium, they found that the expression of inflammatory cytokines such as TNF-*α*, IL-1*β*, and IL-6 reduced, myocardial apoptosis decreased and the left ventricular function improved significantly.

### 7.5. Multigene Modified MSCs Exploration

The ideal intervention therapy for heart damage should at least include the survival of transplanted cells and myocardial reperfusion. The effect of single gene is limited. Some researchers were exploring a joint approach to meet different demands of therapy by applying to a variety of genes combination, and they have made some achievements. Yau et al. [62] used MSCs which were transfected by VEGF and IGF-1 plasmid to treat myocardial infarction. They found that the MSCs with combined genes had the highest survival rate after one week, while its improvement in cardiac function also showed the best condition after 3 weeks. All suggest that multigene combination therapy can play a therapeutic effect of the superposition. Some other studies have applied both Ang-1 and Akt to modify MSCs transplantation cooperatively to treat myocardial infarction to meet the demands of angiogenesis and antiapoptosis. They also achieved the expected results. The cardiac function of rats has been further improved, and having shown the long-term therapy effects, the results lasted for 3 months [63]. But the effect of mutigene transfection to biological behavior of stem cells is not clear. Is there a single gene that can have both of the functions in the meantime? Some researchers think that HGF has the ability in angiogenesis, anti-apoptosis, and promoting cell migration, but there is still no acknowledged best gene modification program existing at present. The tissue engineering research of genetic enhancement with MSCs as the carrier has brought new hope to repair damaged hearts.

## 8. Conclusion

Although in some clinical trials stem cells have achieved effectiveness for ischemic heart disease, the effectiveness of stem cells is still lacking of consistency conclusion. Clinical trials should try to unify stem cells separation, cultural method, and transplantation approach, to set reasonable control, and to have enough followup time. The design destination should go beyond the alternative indicators, so as to obtain sufficient convincing evidence to clarify the differences in clinical endpoints, such as survival rate, hospitalization rate, recurrent myocardial infarction. Stem cell transplantation brings about hope, but the study of stem cell therapy in cardiac repair is still in the initial stage. We should be cautious about the study and application of this technology. Improving the survival rate after stem cell transplantation and prompt homing of stem cells may be effective strategies for stem cell therapy.

##  Conflict of Interests

None of the authors has a conflict of interests to declare.

## Figures and Tables

**Table 1:** Randomized controlled bone marrow-derived cell trials in myocardial infarction.

Studies	Mean age (years)	Patients randomized (patients followup)	LVEF baseline (%)	Followup (month)	Dose	Assessment method	Outcome
Chen et al. (2004) [8]	58	69 (69)	49	6	6 × 10^10^	Echo	EF increased 18%
REPAIR-AMI (2006) [9]	56	204 (187)	47.6	12	2.4 × 10^8^	Angiography	EF increased 2.5%
ASTAMI (2006) [10]	57.4	100 (100)	46.3	6	8.7 × 10^7^	Echo/SPECT/MRI	No effect
TCT-STAMI (2006) [11]	58.5	20 (20)	56	6	4 × 10^7^	Echo/SPECT	EF ↑ 6.7%
BOOST (2009) [12]	56.3	60 (60)	50.7	60	2.5 × 10^9^	MRI	No effect
Janssens et al. (2006) [13]	58.7	67 (66)	47.7	4	1.7 × 10^8^	MRI	No effect
Meluzín et al. (2006) [14]	55	66 (66)	41.7	3	10^8^/10^7^	SPECT/Echo	EF increased 3% (10^8^). No effect (10^7^)
Huikuri et al. (2008) [15]	59.5	80 (77)	60.5	6	3.6 × 10^8^	Echo/Angiography	EF increased 4% (Echo)/7.1% (angiography)
Plewka et al. (2009) [16]	56	60 (56)	37	6	1.44 × 10^8^	Echo	EF increased 10%
REGENT (2009) [17]	57	200 (199)	37	6	1.78 × 10^8^/1.9 × 10^6^ (sorted)	MRI	EF increased 3%
Wöhrle et al. (2010) [18]	61	42 (42)	54	6	3.81 × 10^8^	MRI	EF increased 5.7%
STAR-heart [7]	59.5	391 (391)	32.83	60	6.6 × 10^7^	Angiography	EF increased 6.2%

All studies demonstrated satisfactory patient matching. EF: ejection fraction; Echo: echocardiography; SPECT: single-photon-emission computed tomography; MRI: magnetic resonance imaging.
